# Active commuting to school, cognitive performance, and academic achievement: an observational study in Dutch adolescents using accelerometers

**DOI:** 10.1186/1471-2458-14-799

**Published:** 2014-08-05

**Authors:** Martin L Van Dijk, Renate HM De Groot, Frederik Van Acker, Hans HCM Savelberg, Paul A Kirschner

**Affiliations:** Welten Institute, Faculty of Psychology and Educational Sciences, Open University of the Netherlands, Valkenburgerweg 177, 6419 AT Heerlen, the Netherlands; Department of Human Movement Sciences, Maastricht University, P.O. Box 616, 6200 MD Maastricht, the Netherlands

**Keywords:** Exercise, Physical activity before school, Accelerometry, Executive functioning, School performance, Youth

## Abstract

**Background:**

The current study examined the associations between active commuting to school, cognitive performance, and academic achievement in Dutch adolescents. In addition, it was explored whether these associations were moderated by sex and mediated by depressive symptoms.

**Methods:**

Students in grades 7 and 9 (N = 270; mean age 13.4 years; 53% boys) were included. Active commuting to school was measured objectively by an ActivPAL3™ accelerometer. Cognitive performance was measured by the d2 Test of attention (key components of executive functioning) and the Symbol Digit Modalities Test (information-processing speed). Academic achievement was determined by the mean of the school grades obtained in Dutch, mathematics and English. Depressive symptoms were self-reported.

**Results:**

Active commuting to school constituted 28% of the total amount of time spent moving per week. Active commuting to school was not significantly associated with cognitive performance and academic achievement, overall. However, active commuting to school was positively associated with performance on the d2 Test of attention in girls (*β =* .17, *p =* .037), but not in boys (*β = −*.03, *p =* .660). The associations were not mediated by depressive symptoms.

**Conclusions:**

The associations between active commuting to school and cognitive performance and academic achievement are weak and might be moderated by sex, while the greatest benefits on cognition due to active commuting to school might be with regard to executive functioning. Future studies might make use of experimental designs, because causal relations between active commuting to school and cognitive performance or academic achievement would provide important implications for both education and public health.

## Background

An increasing amount of literature investigates the correlations between active commuting to school (i.e. walking, cycling and other forms of non-motorised transportation) and health outcomes. For example, active commuting to school is positively associated with cardiovascular fitness [1] and negatively associated with overweight [2]. In addition, active commuting to school is an opportunity to increase physical activity levels [3]. Physical activity in turn has been shown to have positive effects on brain structures [4] as well as on cognitive performance [5] and academic achievement [6] in adolescents. As a result, habitual active commuting to school activity may be positively associated with physical activity levels, and consequently cognitive performance and academic achievement in adolescents. Furthermore, active commuting to school can be considered as an acute bout of exercise immediately before the start of school. An acute bout of exercise may immediately increase several cognitive functions [7], which may enhance academic achievement [8]. Therefore, active commuting to school may have acute positive effects on daily cognitive performance at school, and consequently long-term academic achievement. Taken together, habitual active commuting to school might have long-term positive effects on cognitive performance and academic achievement by increasing physical activity levels, as well as long-term positive effects on academic achievement by increasing daily cognitive performance at school.

To the best of our knowledge, no study so far investigated the association between active commuting to school and academic achievement, while only one study has examined the association between active commuting to school and cognitive performance in adolescents [9]. In this study conducted by Martinez-Gomez et al. [9], active commuting to school was measured subjectively by self-report, which has several limitations [10]. Therefore, we used an objective instrument to measure active commuting to school and investigated the associations with both cognitive performance and academic achievement in Dutch adolescents.

Martinez-Gomez et al. [9] found that self-reported active commuting to school was positively associated with cognitive performance in Spanish adolescent girls, but not in boys. In addition, girls who reported spending more than 15 minutes in active commuting to school per day had better cognitive scores than those who spent less than 15 minutes in daily active commuting to school. The authors suggested that this sex-specific effect might be caused by brain-derived neurotrophic factor levels, which are negatively associated with depressive symptoms (improved psychological well-being) in female, but not in male, mice [11]. As physical activity increases brain-derived neurotrophic factor levels [12], physical activity may decrease depressive symptoms (i.e. improve psychological well-being) in girls, but not in boys. Moreover, as depressive symptoms are negatively associated with cognitive performance [13] and academic achievement [14] in adolescents, physical activity may have beneficial effects on cognitive performance and academic achievement in adolescent girls, but not in boys. In addition, adolescent girls report higher levels of depressive symptoms than adolescent boys [15]. Therefore, the beneficial effects of physical activity on depressive symptoms, and consequently cognitive performance and academic achievement, might also be greater in girls than in boys. As a result, the association between active commuting to school and psychological well-being might be greater in girls than in boys, resulting in a positive association between active commuting to school and cognitive performance in girls, but not in boys [9].

In this study, we investigated the associations between habitual active commuting to school and cognitive performance and academic achievement in Dutch adolescents. In addition, we explored whether these associations were moderated by sex and mediated by depressive symptoms. We measured active commuting to school objectively using an ActivPAL3™ accelerometer. Results were controlled for covariates related to physical activity and cognitive performance or academic achievement, such as sex, ethnicity, socioeconomic status (SES), and body mass index (BMI) [5, 16, 17]. We hypothesised positive dose–response associations between active commuting to school and cognitive performance and academic achievement, in accordance with the findings of Martinez-Gomez et al. [9]. In addition, on the basis of the executive function hypothesis, we expected to observe the greatest cognition improvements in the area of executive functioning [18]. Finally, the greatest improvements in academic achievement due to physical activity have previously been found in mathematics [19], so we also separately analysed the association between active commuting to school and mathematics performance.

## Methods

### Study design and participants

Data were gathered as part of The GOALS (Grootschalig Onderzoek naar Activiteiten van Limburgse Scholieren [Large-scale Research of Activities of Limburgs Students]) Study, which was primarily designed to investigate the associations between physical activity and cognitive performance and academic achievement in adolescents. This cross-sectional study was conducted at a secondary school in Heerlen, The Netherlands. Data collection took place from October 2011 to March 2012. All students (N = 526) in grade 7 and grade 9 of senior general secondary education or university preparatory education level were invited to participate. The local Ethical Committee of the Open University (reference number: U2013/07405/HVM) of the Netherlands approved the study.

A sample of 440 students (83.7%) was willing to participate. Of these, 8 were excluded from the analyses because of health or concentration problems, 8 because of injuries or illnesses throughout the entire week, and 8 due to measurement failures of the accelerometer during the data collection week. Moreover, 146 participants did not wear the accelerometer on at least three valid weekdays and were therefore also excluded, leaving a total of 270 students in the analyses.

### Procedures

Before the start of the study, information regarding its background, goals and procedures was distributed to students in the selected classes. An invitation for an informative presentation on the study was attached for the students’ parents/guardians. All students in the selected classes participated in the study, unless their parents/guardians signed an objection form or they themselves had serious objections to doing so. No written informed consent for participation in the study was obtained from the students or their parents/guardians, which was decided in consultation with the Ethical Committee. The Ethical Committee argued that this observational study did not impact health, the school programme or academic achievement of the students, and therefore decided that an informed consent was not necessary. In addition, the participation rate of 83.7% of the invited students indicates that both parents/guardians and students also argued that this study had no significant impact. As far as possible, data were collected by the same research assistants, who were trained in the standard protocols and were given standardised instructions to use.

During a normal gym class, accelerometers were taped on the midpoint of the anterior part of the right thigh of the participants using Tegaderm™ (3M, St. Paul, MN) transparent film roll. The participants were asked to wear the device continuously for one full week, 24 hours/day. To increase compliance, participants were allowed to shift the device to the left thigh when it irritated their skin. Prior to the study, an almost perfect Pearson’s correlation (r = .997, *p* < .001) in number of accelerometer steps between the left and right thigh was found in a pilot study conducted in 23 students wearing an ActivPAL3™ accelerometer on both legs for one complete day. After attaching the accelerometers, participants took a 20-m shuttle-run test and then completed several questionnaires. They were also asked to keep a diary during the full week, in which they recorded their active commuting to school activities as well as relevant details, such as problems with the accelerometer, illness or injuries. At week 2, exactly 1 week later, the participants completed two neuropsychological tests; the d2 Test of attention and the Symbol Digit Modalities Test. Finally, the participants returned their accelerometers and diaries. After completing the study, participants received a gift voucher to the value of 15 euro in return for full participation. At the end of the academic year, school grades throughout that year were provided by the school.

### Instruments

#### Active commuting to school

The ActivPAL3™ accelerometer (Paltechnologies, Glasgow, UK) was used to measure habitual active commuting to school activity. This small device (53 × 35 × 7 mm) was attached at the right thigh of the participants and measured the movements of the upper leg during a variety of activities, such as walking and cycling to school. Data were recorded at 20 Hz and summarized in 15-second time intervals (epochs). Data output for each epoch included number of steps, energy expenditure (metabolic equivalent/h), and time spent sitting/lying, standing, and moving.

**Figure 1:**
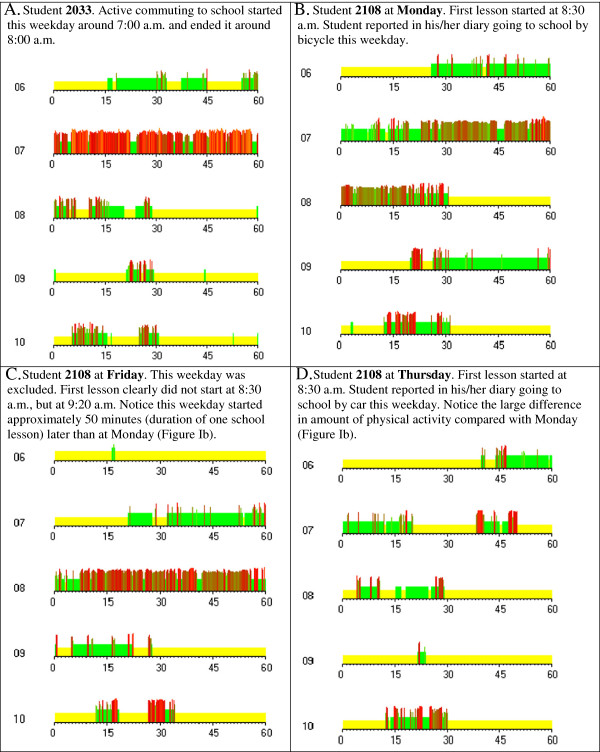
**Daily overviews between 6:00 a.m. and 11:00 a.m. of the accelerometer data.** Yellow colour represents sitting/lying, green colour represents standing, red colour represents physical activities, such as walking or cycling to school. Higher and darker lines illustrate higher amount of physical activity.

The total amount of moving time (i.e. minutes spent moving) on weekdays between 7:00 a.m. and 8:40 a.m. was used as measure of habitual active commuting to school activity. This period was representative for active commuting to school activity, because the students reported, except of active commuting to school, no other physical activities before school. In addition, the ActivPAL3™ accelerometer daily overviews showed that active commuting to school contained almost the entire amount of moving time before school, see for examples Figure 1a-d. Starting point of 7:00 a.m. was chosen, because the accelerometer daily overviews showed that active commuting to school started at 7:00 a.m. at the earliest (see Figure 1a, for example), so all the active commuting to school activity was included. The students’ first lesson of the school day started at 8:30 a.m.; however, to prevent an underestimation of active commuting to school activity in students arriving at school too late, 8:40 a.m. was used as cut-off point of the active commuting to school period.

Habitual active commuting to school activity was estimated on a minimum of three valid weekdays when the student wore the accelerometer the whole day (24 hours/day). Because the accelerometer was taped at the thigh of the students, non-wear time was not an issue. Nevertheless, we used the weekly overviews of the accelerometer data to determine whether the students removed the accelerometer during the week, a detailed description has been described elsewhere [5]. Weekdays were included when a student’s first lesson began with the first period of the school day (i.e., 8:30 a.m.). The school timetable, students’ diaries (filled in during the data collection week) and accelerometer daily overviews were used to determine whether a student’s first lesson started at 8:30 a.m. Using the accelerometer daily overviews, weekdays were removed when the accelerometer data clearly showed that a student’s first lesson did not start at 8:30 a.m. (see Figure 1b compared with Figure 1c, for example). Weekdays were not removed when it was clear from the accelerometer daily overviews that a student’s first lesson started at 8:30 a.m., despite large differences in active commuting to school activity for the same student compared with other weekdays. Large within-subject weekday differences were possible, for example because some students went to school by bus or car one weekday and by bicycle another weekday (see Figure 1b compared to Figure 1d, for example). Because of the large within-subject weekday differences, we decided that at least three valid weekdays had to be available to determine active commuting to school, according to previous research of Borrestad et al. [20]. In addition, paired sample t-tests showed no significant differences (*p* > .05) in active commuting to school activity between the weekdays, therefore we concluded that three random weekdays were appropriate for determining active commuting to school.

Finally, to estimate the total amount of habitual active commuting to school activity per week, the total amount of time spent active commuting to school was computed assuming a round-trip, according to research of Tudor-Locke et al. [21]. As a result, the mean moving time on weekdays between 7:00 a.m. and 8:40 a.m. was multiplied by five (the number of weekdays per week) and multiplied by two (to compute a round-trip). In formula: habitual active commuting to school activity = mean moving time (minutes) on weekdays between 7:00 a.m. and 8:40 a.m. * 5 (weekdays) * 2 (round-trip).

#### Cognitive performance

The d2 Test of attention [22] was selected as measure of response inhibition [23] and selective attention [24], both key components of executive functioning [25]. The d2 Test of attention is a widely used neuropsychological test and the construct validity has been well supported in several European samples [24]. The d2 Test of attention consists of 14 rows, each with 47 interspersed ‘p’ and ‘d’ characters. The characters had one to four dashes configured individually, or in pairs, above and/or below each letter. The target symbol was a ‘d’ with two dashes (hence ‘d2’), regardless of whether or not both the dashes appeared above the ‘d’, both appeared below the ‘d’, or one appeared above and one appeared below the ‘d’. The participants’ task was to cross out as many target symbols as possible, moving from left to right, with a limit of 20 seconds/row. No pauses were permitted. An overall performance score was calculated by the total number of correctly crossed-out symbols minus the total number of incorrectly crossed-out symbols.

The Symbol Digit Modalities Test [26] was selected as measure of information-processing speed. The Symbol Digit Modalities Test is widely used in many studies in children and adolescents and has shown good reliability (ICC > 0.87) in young adults [27]. This test contains nine numbers coupled with nine symbols in a random order on the top of a page. On the remainder of the page, boxes are presented with symbols only. Participants were asked to fill in as many corresponding numbers as possible within 90 seconds. The total number of digits correctly coupled to symbols was scored.

#### Academic achievement

The school provided school grades (ranging from 1.0 = very bad, to 10.0 = outstanding) at the end of the academic year. The mean of the school grades in Dutch (native language), mathematics and English as a modern foreign language was used as measure of academic achievement. We expected to observe the greatest effects of active commuting to school on mathematics performance, as this is significantly correlated with all measures of executive functioning [28]. Therefore, the mean school grade for mathematics was analysed separately as an indication of executive functioning.

#### Depressive symptoms

The Center for Epidemiologic Studies of Depression Scale (CES-D) was selected as measure of experience of depressive symptoms [29]. Recent research has shown that the CES-D is an appropriate instrument for measuring depression in adolescents, with an internal consistency (Cronbach’s alpha) ranging from .78 to .82 [30]. In this 20-item self-report scale, students rated the frequency of 20 depressive symptoms over the previous week. A total severity score was calculated by summing all items and ranged from 0 (not depressed) to 60 (high number of depressive symptoms).

#### Covariates

The dichotomous variables of sex (coded as −0.5 = boys, 0.5 = girls), academic year (coded as −0.5 = grade 7, 0.5 = grade 9), school level (coded as −0.5 = senior general secondary education, 0.5 = university preparatory education), and ethnicity (coded as −0.5 = native Dutch [both parents/guardians had been born in The Netherlands] and 0.5 = non-native Dutch) were centred. The highest educational level of the parents/guardians was used as a proxy of SES [31]. If the parents/guardians had a secondary vocational education at most, SES was coded as ‘low-medium’ = *−*0.5, in all other cases SES was coded as ‘high’ = 0.5, following the classification of the Dutch Ministry of Public Health, Welfare and Sport [32].

Weight in kg and height in m (to 2 decimal places) were measured by one of the researchers. BMI was then calculated by dividing weight by height squared.

Time spent moving per week corrected for active commuting to school was calculated by subtracting time spent active commuting to school from total time spent moving per week. Total time spent moving per week was measured by the ActivPAL3™ accelerometer and based on at least 4 valid days with complete accelerometer data (24 hours/day) including both weekend days. A detailed description of the methodologies used to calculate the total amount of physical activity per week have been previously reported [5].

### Data analysis

Analyses were performed with SPSS for Windows (version 19.0; SPSS Inc., Chicago, Illinois). The level of significance was .05 in all analyses. Sex differences were analysed by independent sample t-tests and Pearson’s Chi-square test for continuous and dichotomous variables, respectively.

Associations between active commuting to school and cognitive performance or academic achievement, controlling for covariates, were analysed by multiple linear regression analyses. Firstly, continuous variables and covariates were transformed into z-scores. Secondly, associations between covariates and cognitive performance or academic achievement were modelled (step A). Thirdly, active commuting to school was added to the model (step B). Fourthly, the potential interaction effect of sex on the associations between active commuting to school and cognitive performance or academic achievement was investigated. Therefore, the centred score of sex was multiplied with the z-score of active commuting to school and then added to the model (step C). Fifthly, significant interaction effects in step C were analysed by simple slopes analyses to further investigate the nature of the statistical interactions. Finally, significant associations between active commuting to school and cognitive performance or academic achievement were analysed by multiple linear regression analyses following the steps of Preacher and Hayes [33] to investigate whether depressive symptoms mediated these associations.

The reported results of the multiple linear regression analyses exclude outliers (>3 standard deviations). The p-p plots were normally distributed. There was no multicollinearity (Pearson’s correlation between covariates < .80) in the multiple regression models.

## Results

The descriptive statistics of the entire study sample are shown in Table 1. Boys (Table 2) had significantly lower scores on academic achievement (independent t-test, *p < .001*) and mathematics performance (*p =* .038), and reported significantly lower levels of depression symptoms (*p <* .001) than did girls (Table 3). In addition, girls performed better on the Symbol Digit Modalities Test (independent t-test, *p = .016*), however when corrected for covariates in the regression model, this difference in performance was not significant anymore (Table 4, step A).

**Table 1:** **Descriptive statistics of the entire study sample (N = 270)**

	Mean ± SD	Frequencies (%)	95% confidence limits
**Age**	13.42 ± 1.28		13.27 – 13.57
**Sex**			
Boys		143 (53.0%)	
Girls		127	
**Ethnicity**			
Native Dutch		248 (91.9%)	
Non-native Dutch		22	
**Academic year**			
Grade 7		136 (50.4%)	
Grade 9		134	
**School level**			
Senior general secondary education		73 (27.0%)	
University preparatory education		197	
**SES**			
Low-medium		65 (24.4%)	
High		201	
**BMI (kg/m** ^**2**^ **)**	19.14 ± 2.70		18.81 – 19.46
**Depressive symptoms**	10.86 ± 8.26		9.86 – 11.86
**Total time spent moving per week (min.)**	834.60 ± 215.21		806.76 – 862.44
**Active commuting to school (min./week)**	230.80 ± 94.75		219.44 – 242.15
**Time spent moving per week corrected for active commuting to school (min.)**	602.90 ± 188.91		578.46 – 627.33
**Active commuting to school as % of total time spent moving per week**	28.15 ± 10.34		26.60 – 30.40
**D2 Test of attention**	175.84 ± 26.66		172.61 – 179.06
**Symbol Digit Modalities Test**	60.11 ± 10.50		58.83 – 61.40
**Academic Achievement**	6.83 ± 0.78		6.73 – 6.92
**Mathematics performance**	6.97 ± 1.10		6.84 – 7.10

SES, socioeconomic status; BMI, body mass index.

**Table 2:** **Descriptive statistics of the study sample of boys (N = 143) and statistical differences with girls**

	Mean ± SD	Frequencies	95% confidence limits	Mean difference with girls (95% confidence intervals)	Effect size (Cohen’s ***d***)
**Age**	13.40 ± 1.31		13.18 – 13.61	-.04	0.03
**Ethnicity**					
Native Dutch		134 (93.7%)			
Non-native Dutch		9			
**Academic year**					
Grade 7		73 (51.0%)			
Grade 9		70			
**School level**					
Senior general secondary education		44 (30.8%)			
University preparatory education		99			
**SES**					
Low-medium		32 (22.9%)			
High		108			
**BMI (kg/m** ^**2**^ **)**	18.98 ± 2.81		18.51 – 19.45	−0.33	0.12
**Depressive symptoms**	9.12 ± 7.21		7.91 – 10.34	−3.63*	0.45
**Total time spent moving per week (min.)**	857.41 ± 222.09		817.60 – 897.21	48.10	0.22
**Active commuting to school (min./week)**	237.23 ± 96.89		221.21 – 253.24	13.68	0.14
**Time spent moving per week corrected for active commuting to school (min.)**	618.38 ± 203.59		581.89 – 654.88	32.66	0.17
**Active commuting to school as % of total time spent moving per week**	28.50 ± 10.60		25.86 – 29.67	0.74	0.07
**D2 Test of attention**	173.27 ± 26.74		168.82 – 177.72	−5.49	0.21
**Symbol Digit Modalities Test**	58.60 ± 10.87		56.74 – 60.46	−3.15*	0.30
**Academic Achievement**	6.62 ± 0.75		6.49 – 6.75	−0.44*	0.59
**Mathematics performance**	6.83 ± 1.11		6.65 – 7.02	−0.28*	0.26

*Statistically significant difference between the sexes at *p < .05.*

SES, socioeconomic status; BMI, body mass index.

**Table 3:** **Descriptive statistics of the study sample of girls (N = 127)**

	Mean ± SD	Frequencies	95% confidence limits
**Age**	13.44 ± 1.25		13.22 – 13.66
**Ethnicity**			
Native Dutch		114 (89.8%)	
Non-native Dutch		13	
**Academic year**			
Grade 7		63 (49.6%)	
Grade 9		64	
**School level**			
Senior general secondary education		29 (22.8%)	
University preparatory education		98	
**SES**			
Low-medium		33 (26.2%)	
High		93	
**BMI (kg/m** ^**2**^ **)**	19.31 ± 2.58		18.86 – 19.76
**Depressive symptoms**	12.76 ± 8.92		11.19 – 14.32
**Total time spent moving per week including active commuting to school (min.)**	809.31 ± 205.36		770.50 – 848.11
**Active commuting to school (min./week)**	223.55 ± 92.13		207.37 – 239.72
**Time spent moving per week corrected for active commuting to school (min.)**	585.72 ± 170.45		553.51 – 617.93
**Active commuting to school as % of total time spent moving per week**	27.76 ± 10.07		26.81 – 29.49
**D2 Test of attention**	178.76 ± 26.37		172.61 – 179.06
**Symbol Digit Modalities Test**	61.75 ± 9.87		58.83 – 61.40
**Academic Achievement**	7.06 ± 0.75		6.73 – 6.92
**Mathematics performance**	7.12 ± 1.08		6.84 – 7.10

SES, socioeconomic status; BMI, body mass index.

**Table 4:** **Multiple linear regression analyses for active commuting to school associated with cognitive performance and academic achievement in adolescents**

	D2 Test of attention		Symbol Digit Modalities Test		Academic achievement		Mathematics performance	
		***β***		***β***		***β***		***β***
**Step A (R** ^**2**^ **)**	.12*		.24*		.23*		.16*	
Sex		.07		.09		.26*		.15*
Ethnicity		.04		.02		.06		.09
Academic year		.31*		.42*		-.25*		-.09
School level		.18*		.15*		.15*		.14*
SES		.02		-.12		.07		0.8
BMI		.03		.14*		-.11		-.17*
Depressive symptoms		-.01		.09		-.15*		-.20*
Time spent moving per week corrected for active commuting to school		.10		.15*		-.06		.01
**Step B (**Δ**R** ^**2**^ **)**	.00		.00		.00		.01	
Active commuting to school		.05		.04		.04		.08
**Step C (**Δ**R** ^**2**^ **)**	.02*		.00		.00		.00	
Active commuting to school* Sex		.13*		.02		.00		.07

*β* = Standardised regression coefficients.

*Statistically significant at *p <* .05.

SES, socioeconomic status; BMI, body mass index.

### Active commuting to school

Students spent 231 minutes per week active commuting to school on average, with no significant difference between boys (237 minutes) and girls (224 minutes; independent t-test, *p =* .237). Active commuting to school contained 28.2% of the total time spent moving per week, with no significant difference between boys (28.5%) and girls (27.8%; independent t-test*, p =* .588).

### Active commuting to school and cognitive performance and academic achievement

Active commuting to school was not significantly associated with performance on the d2 Test of attention, the Symbol Digit Modalities Test, academic achievement and mathematics achievement, overall (Table 4, step B). A significant interaction between active commuting to school and sex on the d2 Test of attention was found (Table 4, step C). Furthermore, simple slopes analyses revealed a significantly positive association between active commuting to school and performance on the d2 Test of attention in girls (*β =* .17, *p* = .037), but no significant association in boys (*β = −*.03, *p =* .660).

### Mediating role of depressive symptoms in the associations between active commuting to school and cognitive performance and academic achievement

Active commuting to school was not significantly associated with self-reported depressive symptoms in girls (*β =* .00, *p* = .970). Therefore, a basic requirement for mediation was not fulfilled. It can be concluded that the association between active commuting to school and performance on the d2 Test of attention in girls was not mediated by self-reported depressive symptoms. In addition, all the other associations between active commuting to school and cognitive performance and academic achievement were not statistically significant. Therefore, all these associations were also not mediated by depressive symptoms, because a basic requirement for mediation was not fulfilled.

## Discussion

The main findings of this study suggest that, in adolescent girls, active commuting to school is positively associated with performance on the d2 Test of attention, which measures key components of executive functioning such as response inhibition and selective attention [23, 24]. In contrast, active commuting to school is not significantly associated with other measures of cognitive performance and academic achievement in both boys and girls. Furthermore, active commuting to school is not significantly associated with self-reported depressive symptoms, which indicates that the associations between active commuting to school and cognitive performance and academic achievement are not mediated by such symptoms. Our results are in accordance with those of Martinez-Gomez et al. [9] who found that self-reported active commuting to school was positively associated with cognitive performance in adolescent girls. Our research expands on the research of Martinez-Gomez et al. [9] by objectively measuring active commuting to school by accelerometry. Moreover, we investigated associations between active commuting to school and both cognitive performance and academic achievement, and investigated whether these associations were mediated by self-reported depressive symptoms.

In girls, active commuting to school was positively associated with performance on the d2 Test of attention, a measure of response inhibition and selective attention, both key components of executive functioning [25]. In contrast, active commuting to school was not significantly associated with performance on the Symbol Digit Modalities Test, a measure of information-processing speed, which is usually not classified as an executive function [25]. These results concur with an increasing amount of literature suggesting that gains in children’s cognitive functioning due to physical activity are most clearly observed in tasks that involve executive functioning (see Tomporowski et al. [34] for a review). Since executive functioning is of crucial importance to success in school [35], this finding might be useful and important for education, although we did not find a significant association between active commuting to school and academic achievement.

The sex-specific association between active commuting to school and executive functioning observed in our study is in agreement with the findings of Kwak et al. [16], who reported a positive association between objectively measured physical activity and academic achievement in adolescent girls, but not in boys. In addition, our results also showed that the associations between active commuting to school and both performance on the Symbol Digit Modalities Test and mathematics performance tended to be more positive in girls than in boys, although those associations were not statistically significant (Table 4, step C). Martinez-Gomez et al. [9] suggested that these sex-specific associations might be explained by depressive symptoms. However, we found no evidence that the associations between active commuting to school and cognitive performance and academic achievement are mediated by self-reported depressive symptoms. Another explanation suggested by Martinez-Gomez et al. [9] might be the higher levels of total time spent moving per week for boys compared to girls. Although many studies in adolescents have reported differences in physical activity levels between boys and girls [36], we found no significant difference in total time spent moving per week between boys and girls (Table 2). On the basis of the results described above, we suggest that other factors may play a more important role in the sex-specific association between active commuting to school and executive functioning observed in our study, as well as that of Martinez-Gomez et al. [9]. The first of these is (school-related) stress, which is negatively associated with cognitive performance and academic achievement in adolescents [37]. Adolescent girls report significantly higher levels of (school-related) stress than boys [38]. Physical activity is associated with lowered stress [39] and might have relatively greater beneficial effects on (school-related) stress in girls (because of their higher stress levels) than in boys. Consequently, active commuting to school (i.e. physical activity before school) might decrease (school-related) stress levels and in turn increase cognitive performance more in girls than in boys, resulting in a positive association between active commuting to school and cognitive performance in girls, but not in boys. The second factor is physical activity, which increases the circulation and production of insulin-like growth factor I [40]. Insulin-like growth factor I and female estrogen interact in the promotion of neuronal survival and neuroprotection [41]. Therefore, this sex-specific hormone might underlie the sex-specific associations between physical activity or active commuting to school and cognitive performance/academic achievement observed in our study and those of Martinez-Gomez et al. [9] and Kwak et al. [16]. We suggest future studies in adolescents, which focus on the effects of physical activity or active commuting to school on cognitive performance/academic achievement, in order to account for the role of (school-related) stress and sex-specific hormones.

The major strength of this study is that active commuting to school was objectively measured by the ActivPAL3™ accelerometer. Previous research has shown that this device is a reliable instrument with which to measure physical activity in adolescent girls [42] and young adults [43]. In addition, as this accelerometer was placed on the thigh, it was accurate in classifying time spent moving during 93.3% of the time spent cycling [44], a common activity in commuting in Dutch adolescents [45]. Also, we objectively measured cognitive performance and academic achievement and controlled for several potential confounders. Finally, 83.7% of the invited students participated in our study and the students included in analyses did not differ in sex, BMI, executive functioning, and academic achievement from students excluded from analyses because of missing accelerometer wear-time. Taken together, in our opinion, these methodological factors ensure that our results are representative of the associations between active commuting to school and cognitive performance and academic achievement in our study sample of Dutch adolescents.

However, our study also has some limitations. We used only one secondary school, which makes it hard to generalise our findings to the entire Dutch adolescent population. Information about the mode of active commuting to school is missing as the ActivPAL3™ accelerometer cannot accurately distinguish between activities, for example distinguish walking from cycling. Because cycling requires a higher intensity than walking [3], this might be an important limitation. Finally, we based the active commuting to school period on the daily overviews of the accelerometer data, however, an objective definition of this period is missing.

## Conclusions

Active commuting to school is positively associated with executive functioning in adolescent girls. In contrast, other associations between active commuting to school and cognitive performance and academic achievement in both boys and girls are not statistically significant. These results indicate that the associations between active commuting to school and cognitive performance and academic achievement are weak and might be moderated by sex, while the greatest benefits on cognition due to active commuting to school might be with regard to executive functioning. We conducted the first observational study in the associations between objectively measured active commuting to school and cognitive performance and academic achievement in adolescents. Causal relations between active commuting to school and cognitive performance or academic achievement would provide important implications for both education and public health; therefore we recommend future studies to use experimental designs.

## Abbreviations

SES: Socioeconomic status
BMI: Body mass index
CES-D: Center for Epidemiologic Studies of Depression Scale.

## References

[1] Cooper AR, Wedderkopp N, Jago R, Kristensen PL, Moller NC, Froberg K, Page AS, Andersen LB (2008). Longitudinal associations of cycling to school with adolescent fitness. Prev Med.

[2] Bere E, Seiler S, Eikemo TA, Oenema A, Brug J (2011). The association between cycling to school and being overweight in Rotterdam (The Netherlands) and Kristiansand (Norway). Scand J Med Sci Sports.

[3] Chillon P, Ortega FB, Ruiz JR, Veidebaum T, Oja L, Maestu J, Sjostrom M (2010). Active commuting to school in children and adolescents: an opportunity to increase physical activity and fitness. Scand J Public Health.

[4] Thomas AG, Dennis A, Bandettini PA, Johansen-Berg H (2012). The effects of aerobic activity on brain structure. Front Psychol.

[5] Van Dijk ML, De Groot RHM, Savelberg HCM, Van Acker F, Kirschner PA (2014). The association between objectively measured physical activity and academic achievement in Dutch adolescents: findings from The GOALS Study. J Sport Exerc Psychol.

[6] Singh A, Uijtdewilligen L, Twisk JW, van Mechelen W, Chinapaw MJ (2012). Physical activity and performance at school: a systematic review of the literature including a methodological quality assessment. Arch Pediatr Adolesc Med.

[7] Davis CL, Tomporowski PD, McDowell JE, Austin BP, Miller PH, Yanasak NE, Allison JD, Naglieri JA (2011). Exercise improves executive function and achievement and alters brain activation in overweight children: a randomized, controlled trial. Health Psychol.

[8] Hillman CH, Kamijo K, Scudder M (2011). A review of chronic and acute physical activity participation on neuroelectric measures of brain health and cognition during childhood. Prev Med.

[9] Martinez-Gomez D, Ruiz JR, Gomez-Martinez S, Chillon P, Rey-Lopez JP, Diaz LE, Castillo R, Veiga OL, Marcos A (2011). Active commuting to school and cognitive performance in adolescents: the AVENA study. Arch Pediatr Adolesc Med.

[10] Slootmaker SM, Schuit AJ, Chinapaw MJ, Seidell JC, van Mechelen W (2009). Disagreement in physical activity assessed by accelerometer and self-report in subgroups of age, gender, education and weight status. Int J Behav Nutr Phys Act.

[11] Monteggia LM, Luikart B, Barrot M, Theobold D, Malkovska I, Nef S, Parada LF, Nestler EJ (2007). Brain-derived neurotrophic factor conditional knockouts show gender differences in depression-related behaviors. Biol Psychiatry.

[12] Marais L, Stein DJ, Daniels WM (2009). Exercise increases BDNF levels in the striatum and decreases depressive-like behavior in chronically stressed rats. Metab Brain Dis.

[13] Hartlage S, Alloy LB, Vazquez C, Dykman B (1993). Automatic and effortful processing in depression. Psychol Bull.

[14] Frojd SA, Nissinen ES, Pelkonen MU, Marttunen MJ, Koivisto AM, Kaltiala-Heino R (2008). Depression and school performance in middle adolescent boys and girls. J Adolesc.

[15] Undheim AM, Sund AM (2005). School factors and the emergence of depressive symptoms among young Norwegian adolescents. Eur Child Adolesc Psychiatry.

[16] Kwak L, Kremers SP, Bergman P, Ruiz JR, Rizzo NS, Sjostrom M (2009). Associations between physical activity, fitness, and academic achievement. J Pediatr.

[17] Sigfusdottir ID, Kristjansson AL, Allegrante JP (2007). Health behaviour and academic achievement in Icelandic school children. Health Educ Res.

[18] Davis CL, Tomporowski PD, Boyle CA, Waller JL, Miller PH, Naglieri JA, Gregoski M (2007). Effects of aerobic exercise on overweight children’s cognitive functioning: a randomized controlled trial. Res Q Exerc Sport.

[19] Howie EK, Pate RR (2012). Physical activity and academic achievement in children: A historical perspective. J Sport Health Sci.

[20] Borrestad LA, Ostergaard L, Andersen LB, Bere E (2013). Associations between active commuting to school and objectively measured physical activity. J Phys Act Health.

[21] Tudor-Locke C, Ainsworth BE, Adair LS, Popkin BM (2003). Objective physical activity of filipino youth stratified for commuting mode to school. Med Sci Sports Exerc.

[22] Brickenkamp R, Zillmer E (1998). The d2 Test of Attention.

[23] Lendt M, Gleissner U, Helmstaedter C, Sassen R, Clusmann H, Elger CE (2002). Neuropsychological outcome in children after frontal lobe epilepsy surgery. Epilepsy Behav.

[24] Bates ME, Lemay EP (2004). The d2 Test of attention: construct validity and extensions in scoring techniques. J Int Neuropsychol Soc.

[25] Alvarez JA, Emory E (2006). Executive function and the frontal lobes: a meta-analytic review. Neuropsychol Rev.

[26] Smith A (2010). Symbol Digit Modalities Test.

[27] Hinton-Bayre A, Geffen G (2005). Comparability, reliability, and practice effects on alternate forms of the Digit Symbol Substitution and Symbol Digit Modalities tests. Psychol Assess.

[28] Bull R, Scerif G (2001). Executive functioning as a predictor of children’s mathematics ability: inhibition, switching, and working memory. Dev Neuropsychol.

[29] Radloff LS (1977). The CES-D Scale: A self-report depression scale for research in the general population. Appl Psychol Meas.

[30] Verhoeven M, Sawyer MG, Spence SH (2013). The factorial invariance of the CES-D during adolescence: are symptom profiles for depression stable across gender and time?. J Adolesc.

[31] Kaplan GA, Keil JE (1993). Socioeconomic factors and cardiovascular disease: a review of the literature. Circulation.

[32] Dutch Ministry of Public Health, Welfare and Sport (2011). Nationale Atlas Volksgezondheid. Sociaaleconomische status Nederlandse beroepsbevolking.

[33] Preacher KJ, Hayes AF (2008). Asymptotic and resampling strategies for assessing and comparing indirect effects in multiple mediator models. Behav Res Methods.

[34] Tomporowski PD, Davis CL, Miller PH, Naglieri JA (2008). Exercise and Children’s intelligence, cognition, and academic achievement. Educ Psychol Rev.

[35] Crone EA, Dahl RE (2012). Understanding adolescence as a period of social-affective engagement and goal flexibility. Nat Rev Neurosci.

[36] Sallis JF, Prochaska JJ, Taylor WC (2000). A review of correlates of physical activity of children and adolescents. Med Sci Sports Exerc.

[37] Forrest CB, Bevans KB, Riley AW, Crespo R, Louis TA (2013). Health and school outcomes during children’s transition into adolescence. J Adolesc Health.

[38] De Vriendt T, Clays E, Maes L, De Bourdeaudhuij I, Vicente-Rodriguez G, Moreno LA, Nagy E, Molnar D, Ortega FB, Dietrich S, Manios Y, De Henauw S, Group HS (2012). European adolescents’ level of perceived stress and its relationship with body adiposity—The HELENA Study. Eur J Public Health.

[39] Haugland S, Wold B, Torsheim T (2003). Relieving the pressure? The role of physical activity in the relationship between school-related stress and adolescent health complaints. Res Q Exerc Sport.

[40] Frystyk J (2010). Exercise and the growth hormone-insulin-like growth factor axis. Med Sci Sports Exerc.

[41] Garcia-Segura LM, Cardona-Gomez GP, Chowen JA, Azcoitia I (2000). Insulin-like growth factor-I receptors and estrogen receptors interact in the promotion of neuronal survival and neuroprotection. J Neurocytol.

[42] Dowd KP, Harrington DM, Donnelly AE (2012). Criterion and concurrent validity of the activPAL professional physical activity monitor in adolescent females. PLoS One.

[43] Maddocks M, Petrou A, Skipper L, Wilcock A (2010). Validity of three accelerometers during treadmill walking and motor vehicle travel. Br J Sports Med.

[44] Steeves J, Bowles H, McClain J, Dodd K, Brychta R, Wang J, Chen K (2014). Comparison of postural classification from thigh-worn Actigraph and activPAL accelerometers under laboratory and free-living conditions. Poster Presented at the ISBNPA Conference (San Diego).

[45] Chinapaw MJ, Slootmaker SM, Schuit AJ, van Zuidam M, van Mechelen W (2009). Reliability and validity of the Activity Questionnaire for Adults and Adolescents (AQuAA). BMC Med Res Methodol.

[CR46] The pre-publication history for this paper can be accessed here:http://www.biomedcentral.com/1471-2458/14/799/prepub

